# Technique for Percutaneous Fluoroscopically Guided G-Tube Placement in a High-BMI Patient

**DOI:** 10.1155/2012/807161

**Published:** 2011-11-10

**Authors:** Irwin M. Best

**Affiliations:** Interventional Radiology, Department of Radiology, Emory University School of Medicine, 1364 Clifton Road NE, Atlanta, GA 30322, USA

## Abstract

Enteral feeding is still the preferred method of nutritional support even in patients with excessive body mass index. Often, this mass poses a hindrance in performing routine procedures. We present a case describing the technique used to safely place a fluoroscopically guided G-tube in a patient with a significant nutritional deficit after repair of a ruptured thoracic aneurysm. Her admission weight was in excess of 180 Kg. However, protracted respiratory insufficiency and mechanical ventilation prolonged her hospital course. The G-tube was successfully placed using a fluoroscopically guided technique. The advantages of such an approach are discussed.

## 1. Introduction

Enteral nutritional is preferred even in patients with high body mass index (BMI) [1]. Yet, excessive (BMI) might limit safe percutaneous access to the stomach when transillumination is not possible. As illustrated, percutaneous fluoroscopically guided gastrostomy (PFG) tube placement can be performed safely in patients with excessive BMI.

## 2. Materials and Methods

**Figure 1:**
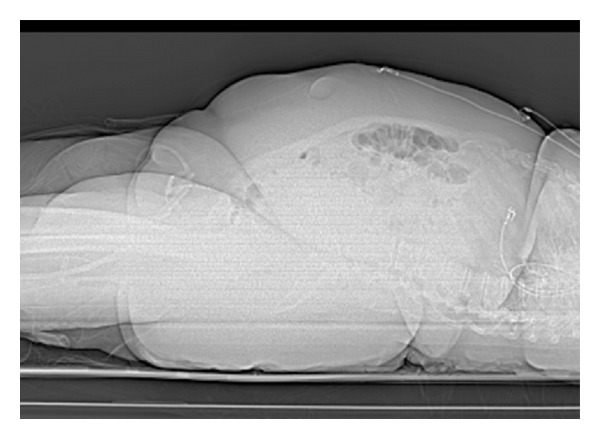
Reconstructed surface view of abdominal and pelvis illustrating patients with high body mass index.

**Figure 2:**
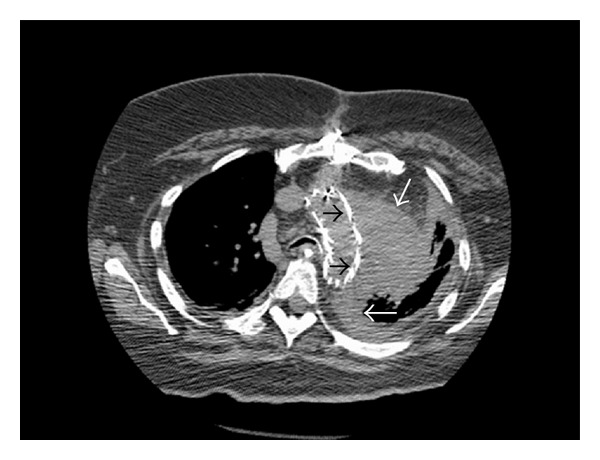
Thoracic stent graft (black arrows) and resolving thoracic hematoma (white arrows).

A PFG-tube was requested in a 53-year-old female with a three-month history of respiratory failure and endograft repair of a ruptured thoracic aneurysm. Patient weighed over 180 Kg at admission. The increased BMI can be seen on the scout images of the initial computed tomogram, Figure 1. Loss of lung volumes and residual hematoma surrounding the stent graft on follow-up CT scan, Figure 2, suggest a need for continued ventilator and nutritional support.

**Figure 3:**
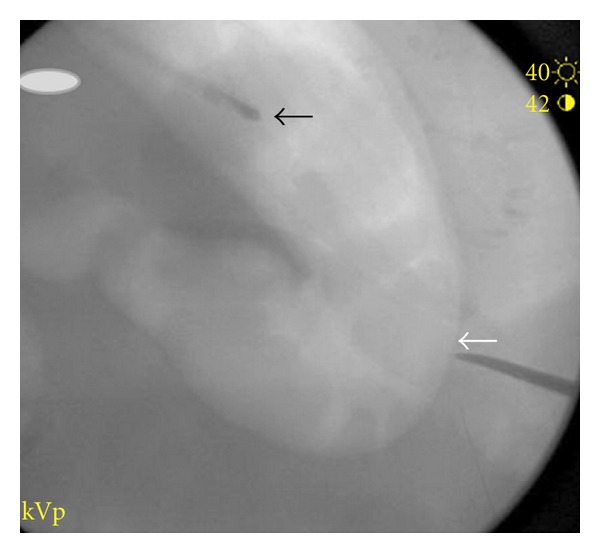
In RAO projection, a hemostat compressed the soft tissue against the insufflated stomach (white arrow) excluding other viscera from the path. The nasogastric tube (black arrow) used for insufflations lies above.

The patient was placed supine. Glucagon, antibiotics, and conscious sedation were administered intravenously. The lower border of the liver was identified with ultrasound and traced on the skin. The stomach was insufflated with air. Bowel was excluded from the access path using multiprojection fluoroscopic imaging while compressing the abdominal wall onto the distended stomach, Figure 3. The access path for the T-fasteners and gastrostomy tube was marked on the skin, and the tract was anesthetized with 10 mL of 2% lidocaine solution. 

**Figure 4:**
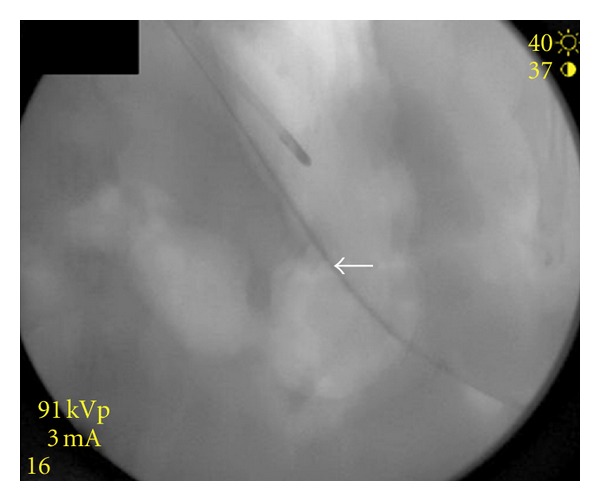
The guide wire (white arrow) directed into the gastric lumen and up the esophagus under fluoroscopic control.

A 16-gauge T-fastener was advanced through the anesthetized path into the stomach. Gastric air was aspirated and contrast was injected to verify access to the stomach. A 180 cm 0.035′′ wire was advanced through the needle and directed up the esophagus beside the feeding tube, Figure 4. The access needle was removed. The T-fastener suture was cut at the skin. A 30 cm 9 French sheath was advanced over the wire into the distal esophagus. The snare was attached to the wire and advanced out the oral cavity as a unit. The guide wire was separated from the snare and removed completely via the mouth. The traction G-tube was locked onto the free end of the snare and pulled into the oral cavity and stomach under fluoroscopic guidance until the flange of the tube rested against the anterior wall of the stomach.

## 3. Results

**Figure 5:**
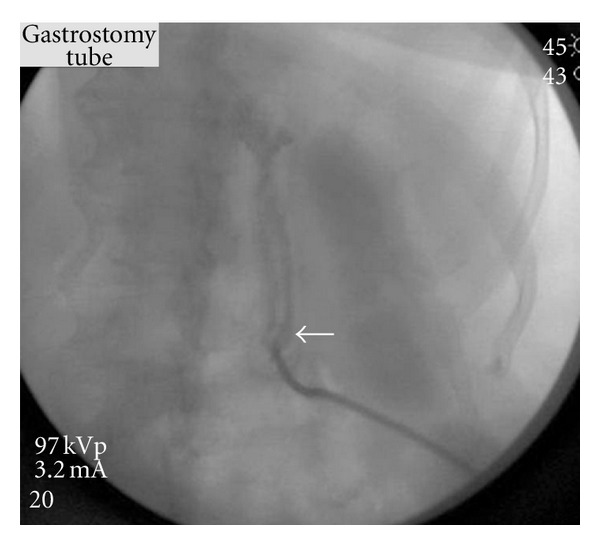
G-tube tip adequately place in stomach (white arrow).

The external bumper was placed at the skin exit site, and length of the traction G-tube was adjusted. The feeding port attachments were then attached. Contrast was injected via the feeding ports, and multiplanar views were obtained to verify good tube location and to rule out any immediate surgical misadventure, Figure 5. Sterile dressings were placed, and the tube was connected to external gravity drainage for 24 hours. The patient was returned to the unit in stable condition. Tube feedings were started after 24 hours and continued without incident. 

## 4. Discussion

Over 283,000 gastrostomy-related services were performed in 2003, 10% of these were performed by radiology service [2]. The important benefits of enteral nutrition have been well established. In their review of the published literature, Gramlich et al. have confirmed that patients have less infectious complications when on enteral feedings [3]. PFG-tube placement has several advantages. Firstly, gastric access for insufflation can be readily obtained with a small feeding tube which the patient can swallow or can be guided into the stomach using fluoroscopy. Where gastric access is difficult because of tumor or other defects, a five French (F) angled catheter and guide wire can be readily passed under fluoroscopy. Secondly, as we have illustrated, fluoroscopy is much better at outlining the distended stomach when transillumination is not possible with the endoscope. Since BMI above 30 is a known risk factor for complications and mortality post tube placement [4], multiplanar imaging must be used to exclude viscera from the intended access path. A steep RAO view of the abdomen or true lateral view is helpful in identifying viscera intestinal components anterior to the stomach. Additionally, very dilute barium given the day before the exam serves to highlight the colon and prevent inadvertent transcolonic peg tube access. Lastly, if attempts to pass the guide wire up the esophagus fail, then the gastric tube can be snared and used to advance the snare to the oropharynx for capture. Lastly, if access to the esophagus proves to be elusive, then two or three T-fasteners might be placed to secure the stomach to the anterior abdominal wall and the balloon-tipped G-tube passed into the stomach with a track developed by a 24 French dilator and peel-away sheath. The gastric balloon is inflated and the peel-away sheath removed.

## 5. Conclusion

Fluoroscopically guided gastrostomy tube placement is a versatile technique that is particularly suited to patients with elevated BMI who are in need of enteral access for long-term nutrition support.
